# Long non-coding RNA PTTG3P functions as an oncogene by sponging miR-383 and up-regulating CCND1 and PARP2 in hepatocellular carcinoma

**DOI:** 10.1186/s12885-019-5936-2

**Published:** 2019-07-24

**Authors:** Qiang Zhou, Wei Zhang, Zhongfeng Wang, Songyang Liu

**Affiliations:** 1 Department of Hepatology, the First Hospital of Jilin University, Changchun, 130021 Jilin China; 2 Department of Hepatobiliary and Pancreatic Surgery, the First Hospital of Jilin University, Changchun, 130021 Jilin China

**Keywords:** Long non-coding RNA, PTTG3P, miR-383, CCND1, PARP2, Hepatocellular carcinoma

## Abstract

**Background:**

Emerging evidence indicates that Long non-coding RNAs (LncRNAs) and microRNAs (miRNAs) play crucial roles in tumor progression, including hepatocellular carcinoma (HCC). However, whether there is a crosstalk between LncRNA pituitary tumor-transforming 3 (PTTG3P) and miR-383 in HCC remains unknown. This study is designed to explore the underlying mechanism by which LncRNA PTTG3P sponges miR-383 during HCC progression.

**Methods:**

qPCR and Western blot were used to analyze LncRNA PTTG3P, miR-383 and other target genes’ expression. CCK-8 assay was performed to examine cell proliferation. Annexin V-PE/PI and PI staining were used to analyze cell apoptosis and cell cycle distribution by flow cytometry, respectively. Transwell migration and invasion assays were used to examine cell migration and invasion abilities. An in vivo xenograft study was performed to detect tumor growth. Luciferase reporter assay and RNA pull-down assay were carried out to detect the interaction between miR-383 and LncRNA PTTG3P. RIP was carried out to detect whether PTTG3P and miR-383 were enriched in Ago2-immunoprecipitated complex.

**Results:**

In this study, we found that PTTG3P was up-regulated in HCC tissues and cells. Functional experiments demonstrated that knockdown of PTTG3P inhibited cell proliferation, migration and invasion, and promoted cell apoptosis, acting as an oncogene. Mechanistically, PTTG3P upregulated the expression of miR-383 targets Cyclin D1 (CCND1) and poly ADP-ribose polymerase 2 (PARP2) by sponging miR-383, acting as a competing endogenous RNA (ceRNA). The PTTG3P-miR-383-CCND1/PARP2 axis modulated HCC phenotypes. Moreover, PTTG3P also affected the PI3K/Akt signaling pathway.

**Conclusion:**

The data indicate a novel PTTG3P-miR-383-CCND1/PARP2 axis in HCC tumorigenesis, suggesting that PTTG3P may be used as a potential therapeutic target in HCC.

**Graphical Abstract:**

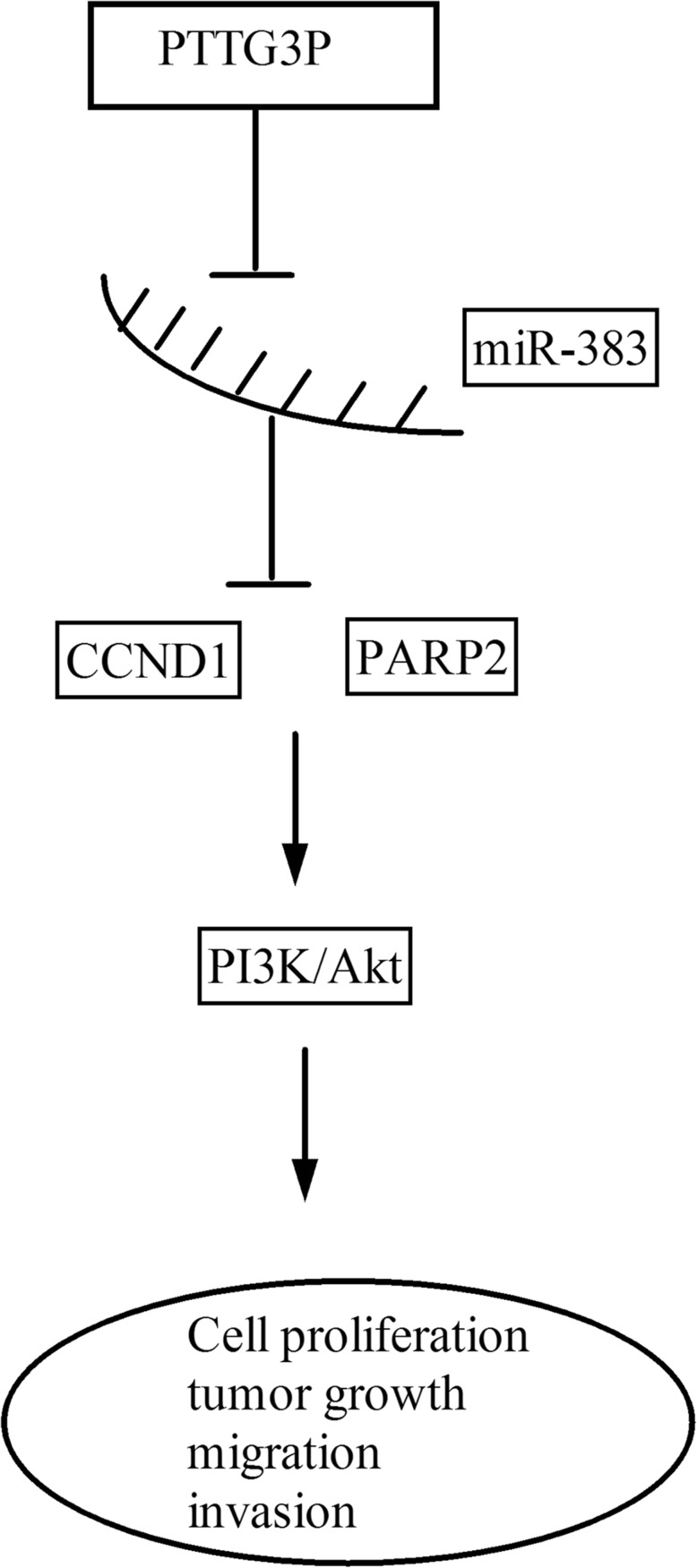

**Electronic supplementary material:**

The online version of this article (10.1186/s12885-019-5936-2) contains supplementary material, which is available to authorized users.

## Background

Hepatocellular carcinoma (HCC) accounts for 90% of liver cancer which is the third cause of cancer-related death worldwide [1, 2]. Despite a variety of advanced therapeutic approaches, including liver resection, chemotherapy, and radiotherapy, or molecular targeted therapy, the prognosis of some HCC is still poor. Thus, it is urgent to understand the molecular mechanism of HCC tumorigenesis to explore novel biomarkers for HCC prognosis, which will promote the development of therapeutic strategy for HCC patients.

Pseudogene, a subclass of long non-coding RNAs (lncRNAs), are considered as genomic loci that resemble real gene, but lost some functionality because they are lack of protein-coding ability because of disabling mutation, lack of transcription, or their inability to encode RNA [3]. However, recent studies have revealed that pseudogene-derived lncRNAs play important roles in cellular process [4–6]. Accumulating evidence indicates that lncRNAs, longer than 200 nucleotides in length and no protein coding potentials, exert crucial roles in pathological process, including cancer development and progression [7, 8]. For example, LincDUSP regulates the colon cancer cell cycle progression and reduces the susceptibility to apoptosis [9], which is upregulated in colon cancer. LncRNA00152 promotes glioma cell proliferation and invasion via the regulation of miR-16, functioning as an oncogene [10]. MicroRNAs (miRNAs) are a family of small non-coding RNA molecules, 22 nucleotides in length, and act as important regulatory modulators of gene expression at the post-transcriptional level through the complete or incomplete base pairs between miRNAs and their targets’ mRNA 3’UTR, resulting in the target mRNA degradation or translational repression [11–13]. MiRNAs are reported to involved in multiple cellular processes [14]. Bioinformatics algorithms including miRCODE (http://www.mircode.org/) suggest that miRNAs can interact with lncRNAs. A series of studies indicate that lncRNAs serve as competing endogenous RNAs (ceRNA) by sponging miRNAs, and modulate the targets of miRNAs [15, 16]. For instance, miR-190 suppresses the EMT of hepatoma cells by targeting lncRNA treRNA [17]. LncRNA SNHG16 promotes the glioma cell proliferation and suppresses cell apoptosis via sponging miR-4518 and upregulating its target RPMI5 [18]. MEG3 inhibits human pancreatic endocrine tumor cell growth and metastasis through acting as a ceRNA of miR-183 [19]. The pseudogene-derived lncRNA PTTG3P has been reported to act as an oncogene in gastric cancer [20] and HCC [21], but the molecular mechanism how PTTG3P interacts with miRNAs in HCC remains poor.

In this study, we found that PTTG3P was upregulated in HCC. Knockdown of PTTG3P suppressed cell growth, migration and invasion, and promoted cell apoptosis by sponging miR-383 and regulating miR-383 targets, CCND1 and PARP2, as well as the PI3K/Akt signaling pathway.

## Methods

### Tissue samples

Fifty paired HCC and adjacent non-tumor tissue samples (within 2 cm of tumor) were acquired from the First Hospital of Jilin University during HCC surgery between January 2015 and July 2018. All tumor tissues were immunohistochemically validated and acquired patients’ consent for the tissues used for this study. The tissues were frozen in liquid nitrogen after resection.

### Cell culture

Six human HCC cell lines, HepG2, Hep3B, Huh-7, HLF, SK-HeP-1, SNU-449, and normal liver cells LO2 were obtained from the Cell Bank of Chinese Academy of Sciences. HepG2, Hep3B, Huh-7, HLF and SK-HeP-1 cells were cultured in Dulbecco’s Modified Eagle Medium (DMEM, HyClone, USA, AC10253739), while SNU-449 and LO2 cells were cultured in RPMI 1640 medium (HyClone, USA, AD12582265). All cells were supplemented with 10% fetal bovine serum (FBS, Coning, USA, 35081003), 100 U/ml penicillin and 100 mg/ml streptomycin. The cells were maintained in a humidified incubator at 37 °C with 5% CO_2_.

### Cell transfection

siRNAs, miR-383 inhibitor, miR-383 mimic, or corresponding controls were acquired from GENECHEM (Shanghai, China), and were transfected into the cells by Lipofectamine™ 3000 according to the manufacturer’s instructions.

Lentivirus overexpressing shRNA against PTTG3P was obtained from GENECHEM (Shanghai, China) and infected HepG2 cells, followed by selection using puromycin.

### RNA isolation and qPCR

Total RNAs were isolated using TriZol reagent (Qiagen) from tissues or transfected cells according to the manufacturer’s protocols. 500 ng of RNA was used for cDNA synthesis using the Prime Script RT regent Kit. The cDNA synthesis of PTTG3P used the random primer and Oligo d(T). The cDNA synthesis of miR-383 used specific miR-383 reverse transcription primer. The fast Start Universal Green Masta (Roche) was used for qPCR on the ABI 7900 detection system. The primers were listed in Table 1. U6 snRNA was used as an internal control to normalize miR-383 expression. GAPDH was used to normalize target genes’ expression. The relative gene expression was normalized to GADPH or U6 expression and calculated according to the 2^−ΔΔCq^ method described by Livak and Schmittgen [22].

**Table 1:** Primers used for RT and qPCR

miR-383 RT primer	5’GTCGTATCCAGTGCGTGTCGTGGAGTCGGCAATTGCACTGGATACGACAGCCAC3’
miR-383	Forward: 5’GGGAGATCAGAAGGTGATTGTGGCT3’
	Reverse: 5’CAGTGCGTGTCGTGGAGT3’
U6 snRNA	Forward: 5’CTCGCTTCGGCAGCACA3’
	Reverse: 5’AACGCTTCACGAATTTGCGT3’
GAPDH	Forward: 5’GGAGCGAGATCCCTCCAAAAT3’
	Reverse: 5’GGCTGTTGTCATACTTCTCATGG3’
PTTG3P	Forward: 5’AAACGAAGAACCAGGCATCCTT3’
	Reverse: 5’GGGAGCATCGAATGTTTTGCC3’
CCND1	Forward: 5’GCTGCGAAGTGGAAACCATC3’
	Reverse: 5’CCTCCTTCTGCACACATTTGAA3’
PARP2	Forward: 5’GCCTTGCTGTTAAAGGGCAAA3’
	Reverse: 5’TCCTTCACACTCCACATGAGCC3’

### Western blot assay

Cells were harvested and lysed using RIPA lysis buffer on ice for 30 min. The lysates were then immunoblotted. The primary antibodies against CCND1 (Rabbit polyclonal to CCND1, 1:1000 dilution, Abcam), and PARP2 (Rabbit polyclonal to PARP2, 1:1000, Abcam) were used. The horseradishperoxidase (HRP)-conjugated anti-rabbit IgG antibody was used as the secondary antibody. Finally, the blots were visualized by an enhanced chemiluminescence system.

### CCK-8 assay

Cell viability was determined using the Cell Counting Kit-8 (CCK-8) according to the manufacturer’s instructions. Briefly, the transfected cells were seeded into the 96-well plate with a density of 3000 cells/well. CCK-8 reagent was added into the medium at 48 h after transfection, followed by incubation for 1 h at 37 °C. Finally, the absorbance was measured at 450 nm by a spectrometer (OD_450 nm_).

### Cell apoptosis assay

Cell apoptosis was analyzed using the Annexin V-PE/PI kit by flow cytometry according to the manufacturer’s protocols. Annexin V-PE (+)/PI (−) represented apoptotic cells, while Annexin V-PE (+)/PI (+) represented the advance apoptotic cells or dead cells.

### Cell cycle analysis

Cell cycle was measured with propidium iodide (PI) by flow cytometry. Briefly, at 48 h after transfection, the cells were harvested and stained with PI using the CycleTest Plus DNA Reagent kit (BD) following the manufacturer’s guide. Finally, the percentage of cells in G0/G1, S and G2/M stages was counted.

### Transwell migration and invasion assays

Cell migration and invasion abilities were analyzed using Transwell Chambers (Corning, 8 μm pore) according to the manufacturer’s protocols. Briefly, the transfected cells (25000 cells) were suspended in 200 μl of serum-free medium and plated into the upper compartment. For the invasion assay, the upper compartment was pre-coated with Matrigel (Sigma). The lower chamber contained 10% FBS-containing medium. After incubation for approximately 20 h, the migrated or invaded cells were fixed and stained by crystal violet. The cells not migrated or invaded into the membrane were scraped using cotton tips.

### Luciferase reporter assay

PTTG3P fragment containing miR-383 binding sites was PCR-amplified and cloned downstream of a luciferase reporter gene in the pmirGLO vector, named pmirGLO-PTTG3P. The pmirGLO-mut PTTG3P (mutations within the binding site) was generated using the Quickchange^XL^ Site-directed Mutagenesis kit (Stratagene) according to the manufacturer’s protocols. The cells were co-transfected with miR-383 and wild-type PTTG3P or mutant PTTG3P vector, together with controls. At 48 h after transfection, the cells were harvested and subjected to Luciferase assay using the Dual-luciferase reporter system (Promega) following the manufacturer’s instructions. The luciferase assays for miR-383 and its targets CCND1 and PARP2 were similar to that of PTTG3P luciferase reporter system.

### In vivo animal study

The animal research was approved by the ethics committee of the First Hospital of Jilin University and the Institutional Animal Care and Use Committee. 4–6 weeks old female nude mice were used for this study. Briefly, 10^6^ cells were suspended in mixture of PBS and Matrigel (1:1) and subcutaneously injected into the flank of the nude mice. The tumor size was measured using a Vernier caliper by the formula V = 1/2 (L*W^2^), where L represented the length (longest dimension), and W represented the width (shortest dimension). After about six weeks, all mice in experiments were euthanized by injection of sodium pentobarbital (100 mg/kg) followed by cervical dislocation. Finally, the xenograft tissues were used for qPCR to analyze PTTG3P expression.

### RNA pull-down assay

RNA pull-down assay was performed using the Pierce™ Magnetic RNA-Protein Pull-Down Kit according to the manufacturer’s instructions. Briefly, HepG2 or Huh-7 cells were transfected with 3’end biotinylated miR-383 or mutant miR-383 or controls treated using Pierce™ RNA 3′ End Desthiobiotinylation Kit, followed by incubation with streptavidin-coated magnetic heads after transfection for 24 h. The level of PTTG3P in the bound fraction was determined by qPCR.

### RNA immunoprecipitation (RIP) assay

RIP assay was performed using a RIP kit (Millipore) according to the manufacturer’s protocols. Briefly, RNAs were isolated and subjected to qPCR. The antibody against Ago2 and negative control IgG were obtained from Abcam (USA).

### Statistical analysis

The data were shown as mean ± standard deviation (SD) from three independent experiments. The differences between groups were analyzed using the Student’s t-test (two groups) or One-way ANOVA (multiple groups) by GraphPad Prism 5.0 software. The value of *P* < 0.05 was considered statistically significant.

## Results

### PTTG3P is highly expressed in HCC tissues and cell lines

**Fig. 1:**
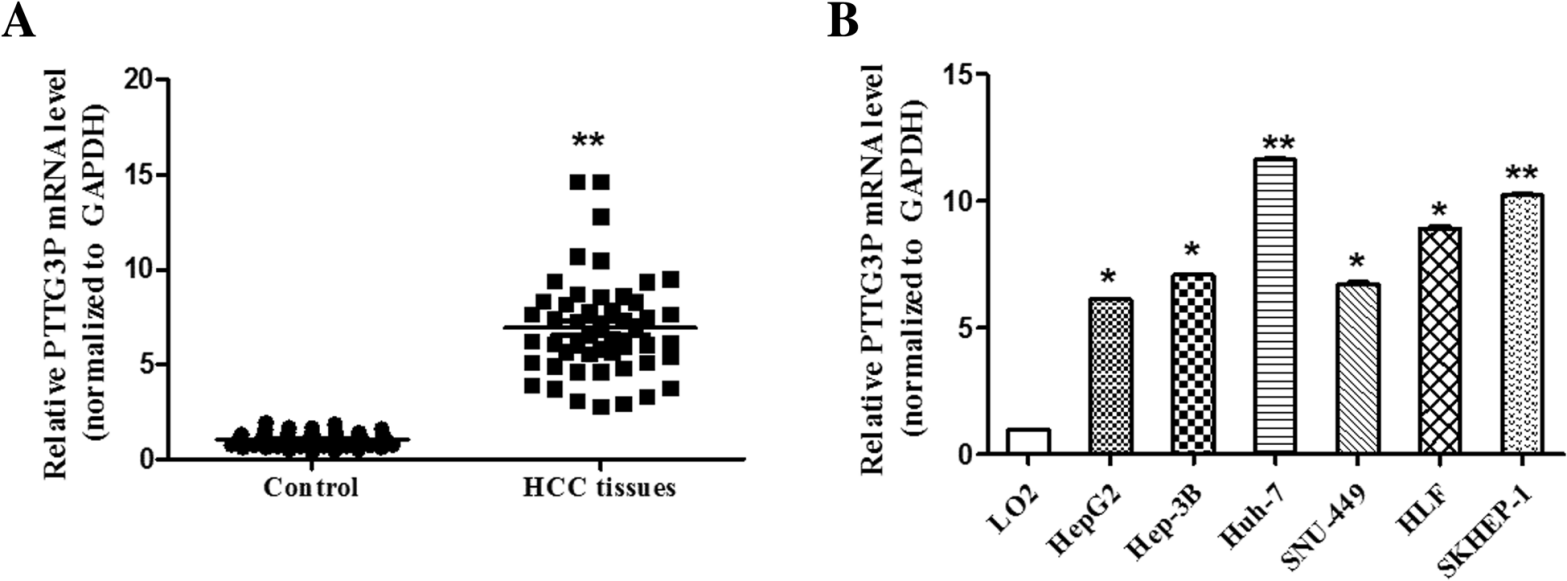
PTTG3P is highly expressed in HCC tissues and cell lines. **a** qPCR analysis of PTTG3P expression in 50 paired HCC tissues and adjacent non-tumor tissues. **b** qPCR analysis of PTTG3P expression in the normal liver cells (LO2) and HCC cells. The data were shown as mean ± SD and the experiment was performed in triplicate. *P < 0.05. **P < 0.01

Firstly, we analyzed the profiles of HCC patients from the Gene Expression Omnibus (GEO) (GSE 76427 and GSE 84402 dataset), and found that PTTG3P was up-regulated in HCC tissues compared to adjacent non-tumor tissues. Next, we aimed to determine whether PTTG3P was overexpressed in HCC. A total of 50 paired HCC tissues were evaluated for PTTG3P expression using qPCR. As results shown in Fig. 1a, PTTG3P was indeed up-regulated in HCC tissues, compared to adjacent non-tumor samples. Moreover, we examined PTTG3P expression in HCC cell lines, Huh-7, HepG2, Hep3B, SNU-449, HLF, SK-Hep-1. PTTG3P was highly expressed in these cell lines, compared to that in the normal liver cells LO2 (Fig. 1b), in which HepG2 and Huh-7 had the lowest and highest levels. Thus, HepG2 and Huh-7 were selected for the following study.

To further investigate the relationship between PTTG3P and clinicopathological features of HCC patients, patients were divided into two groups: high and low PTTG3P levels. As data shown in Table 2, high PTTG3P level was related to tumor size, high stage and metastasis. However, PTTG3P level was not related to other factors, such as age and genders.

**Table 2:** Relationship between the clinicopathological features of HCC and PTTG3P levels

Characteristics	High	Low	P
Gender			
Male	14	13	> 0.05
Female	12	11	
Age			
> 55	11	15	
< =55	12	12	> 0.05
Tumor size (cm)			
< =5	5	13	
> 5	25	7	< 0.001
Stage			
I-II	8	17	
III-IV	21	4	< 0.001
Metastasis			
Negative	2	15	
Positive	29	4	< 0.001

### Knockdown of PTTG3P inhibits cell proliferation and promotes cell apoptosis

**Fig. 2:**
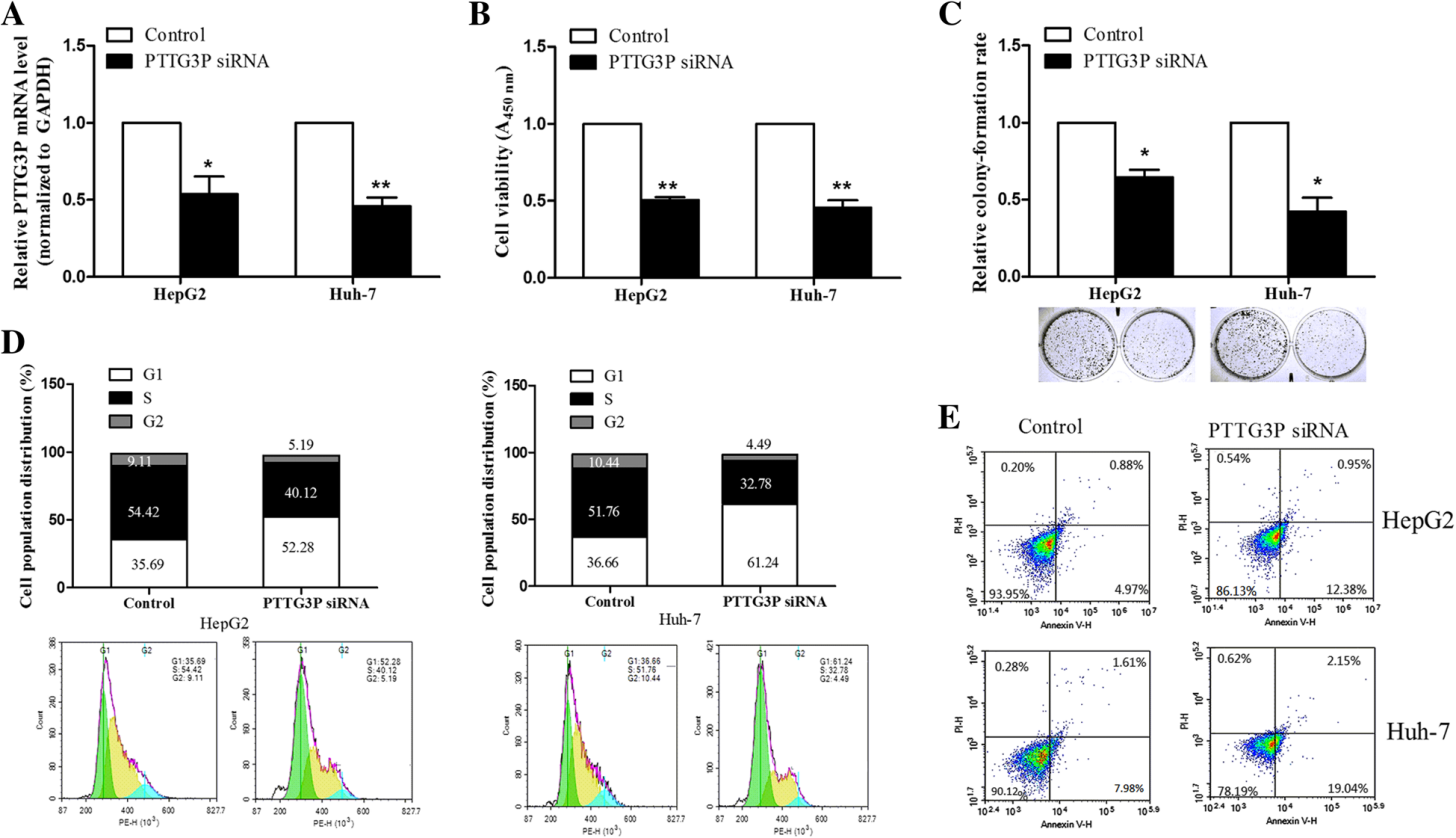
Knockdown of PTTG3P suppresses cell proliferation and promotes cell apoptosis. **a** qPCR analysis of PTTG3P expression in PTTG3P siRNA-transfected HepG2 and Huh-7 cells. **b** CCK-8 assay was used to detect the viability of PTTG3P siRNA in HepG2 and Huh-7 cells. **c** Colony formation assay was performed to detect the colony-forming ability of PTTG3P siRNA in HepG2 and Huh-7 cells. Colonies were counted and captured. **d** Flow cytometry was performed to investigate the cell cycle distribution of HepG2 and Huh-7 cells. The color code represented the G1 stage, S stage and G2 stage, respectively (Left to right). **e** Flow cytometry was performed to detect the apoptosis of HepG2 and Huh-7 cells. LR, early apoptotic cells; UR, late apoptotic cells or dead cells. The results were shown as mean ± SD from three independent experiments and one of the representative results was shown. Each experiment was performed in triplicate. *P < 0.05. **P < 0.01

Next, we aimed to explore the biological functions of PTTG3P in cell proliferation. HepG2 and Huh-7 cells were transfected with PTTG3P siRNA, and the efficiency of siRNA was confirmed by qPCR (Fig. 2a). CCK-8 and colony formation assays were preformed to detect cell proliferation. As results shown in Fig. 2b, knockdown of PTTG3P inhibited cell viability, compared to control group. Similar results were obtained in colony formation assay. PTTG3P knockdown suppressed the colony-forming ability (Fig. 2c). To further investigate whether the effect of PTTG3P on cell proliferation was related to cell cycle progression, we performed flow cytometry to analyze cell cycle. As shown in Fig. 2d, HepG2 and Huh-7 cells transfected with PTTG3P siRNA had a higher percentage of cells in G0/G1 stage, and a lower percentage of cells in S stage, suggesting that PTTG3P knockdown inhibited cell cycle G1/S transition. Finally, cell apoptosis assay showed that cells with PTTG3P siRNA had a high percentage of apoptosis, compared to control group (Fig. 2e, Additional file 2: Figure S2). Overall, these data indicate that PTTG3P knockdown inhibits cell cycle progression, cell proliferation and promotes cell apoptosis.

### Knockdown of PTTG3P inhibits cell migration and invasion

**Fig. 3:**
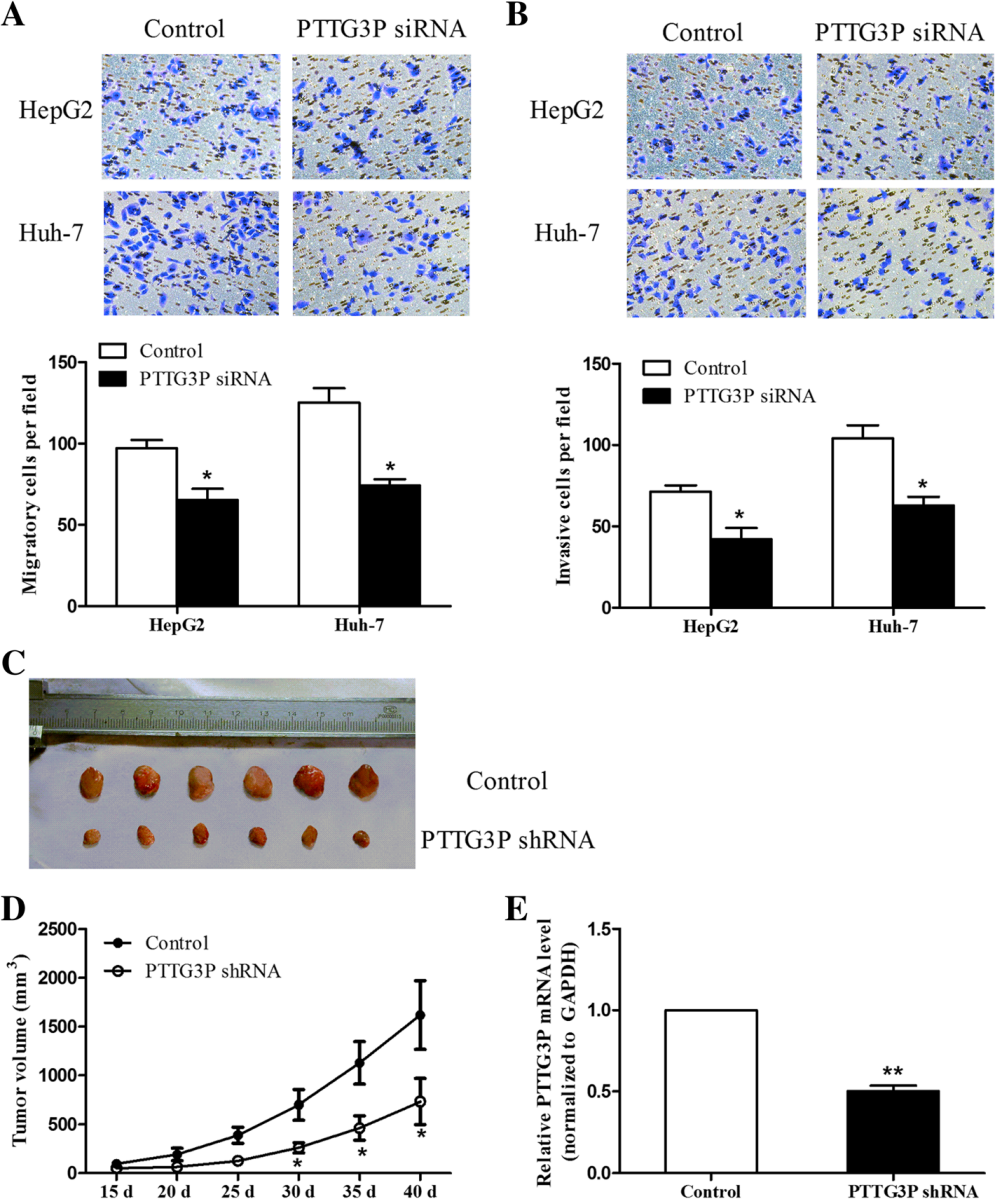
Knockdown of PTTG3P suppresses the cell migration, invasion and tumor growth in vivo. **a**, **b** Transwell assays were preformed to detect the migratory (**a**) and invasive (**b**) abilities of PTTG3P siRNA-transfected or siRNA-control-transfected HepG2 and Huh-7 cells. Migratory and invasive cells were counted and captured. The results were shown as mean ± SD from three independent experiments and one of the representative results was shown. Each experiment was performed in triplicate. **c** HCC cells infected with shRNA-PTTG3P or control were injected into nude mice, respectively. Tumor xenografts in sh-PTTG3P group were smaller than the control group. **d** Tumor volumes were measured after injection every week. **e** qPCR analysis of PTTG3P expression in xenograft tumors. *P < 0.05. **P < 0.01

Considering that metastasis is an important factor involved in tumor progression, we aimed to investigate the effect of PTTG3P on cell migration and invasion. As results shown in Fig. 3a, we found that cells transfected with PTTG3P siRNA had a lower migratory number, compared to control group. Similar results were obtained in the invasion assay which showed that PTTG3P siRNA suppressed the number of invasive cells (Fig. 3b). Taken together, the data indicate that knockdown of PTTG3P exerts tumor suppressive roles in inhibiting cell migration and invasion.

### Knockdown of PTTG3P inhibits HCC tumor growth in vivo

We finally examined the effect of PTTG3P on tumorigenesis in a xenograft study. HepG2 cells stably overexpressing PTTG3P shRNA were subcutaneously injected into the flank of the nude mice. Tumor sizes were measured every week. As shown Fig. 3c and d, we found that the xenograft formed by PTTG3P shRNA-treated cells had a smaller tumor size compared to control group. We also analyzed the expression of PTTG3P in the xenografts to confirm the delivery of PTTG3P shRNA into the mice. The results from the Fig. 3e showed that PTTG3P was downregulated in the xenografts formed by the PTTG3P shRNA transfected cells.

### PTTG3P could directly target miR-383 in HCC cells

**Fig. 4:**
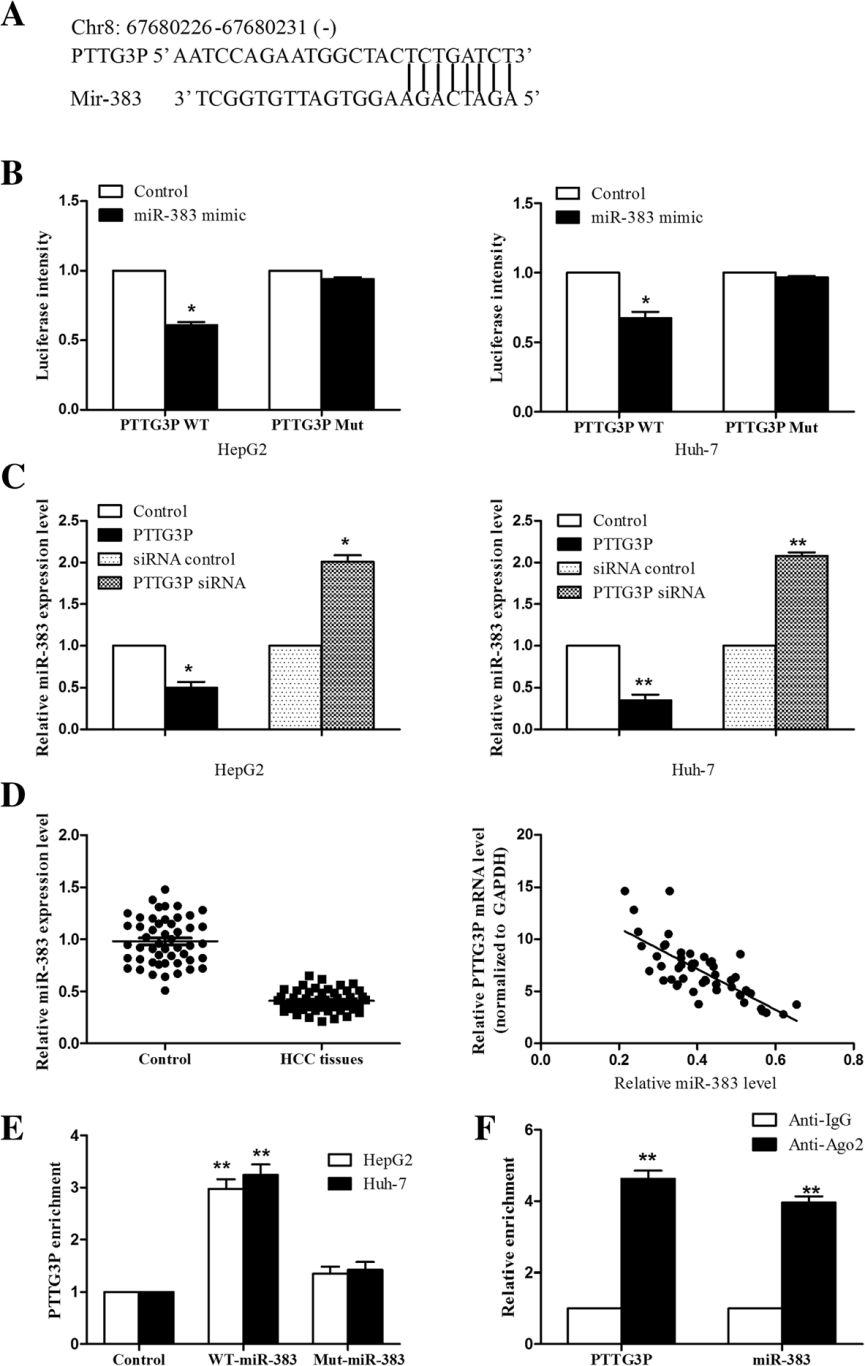
PTTG3P directly interacts with miR-383 in HCC cells. **a** The alignment between PTTG3P and miR-383. **b** Luciferase reporter assay was performed to detect the effect of miR-383 on luciferase intensity controlled by PTTG3P or mutant PTTG3P. **c** qPCR was used to analyze miR-383 expression in HepG2 and Huh-7 cells transfected with PTTG3P, or PTTG3P siRNA or controls. **d** miR-383 expression was analyzed by qPCR in 50 paired HCC tissues and adjacent non-tumor tissues. In addition, there was a negative correlation between miR-383 and PTTG3P expression in HCC. **e** RNA pull-down assay was performed to determine the interaction between PTTG3P and miR-383 in HCC cells. The biotinylated miR-383 or mutant miR-383 was transfected into HCC cells, and PTTG3P expression was analyzed by qPCR. **f** Anti-Ago2 RIP was used to detect the miR-383 and PTTG3P enrichment in immunoprecipitates in HCC cells. IgG was used as a negative control. The results were shown as mean ± SD from three independent experiments and one of the representative results was shown. Each experiment was performed in triplicate. *P < 0.05. **P < 0.01

Accumulating evidence demonstrates that LncRNAs can function as competing endogenous RNAs (ceRNAs) or sponge in modulating the biological functions of miRNAs [23]. Using the bioinformatics algorithms miRCODE (http://mircode.org/), we found that there existed potential interactions between PTTG3P and miR-383 (Fig. 4a). Luciferase reporter assay was performed to determine the interaction between PTTG3P and miR-383. As shown in Fig. 4b, the results indicated that miR-383 reduced the luciferase intensity controlled by PTTG3P, while miR-383 did not affect the luciferase intensity of mutant PTTG3P, compared to the control group. Similar results were shown in Huh-7 cells and HepG2 cell. Moreover, we found that PTTG3P overexpression suppressed miR-383 expression level, while PTTG3P knockdown increased miR-383 level (Fig. 4c). Finally, miR-383 levels were examined in 50 paired HCC tissues and adjacent non-tumor tissues. As shown in Fig. 4d, miR-383 was downregulated in HCC tissues. Importantly, there existed a negative correlation between miR-383 and PTTG3P levels. Finally, we performed RNA pull-down assay to validate the interaction between PTTG3P and miR-383. As shown in Fig. 4e, RNA pull-down assay demonstrated that PTTG3P was more enriched in miR-383 compared to that in mutant miR-383 with broken PTTG3P binding site. In addition, RIP assay indicated that both PTTG3P and miR-383 were enriched in Ago2-containg miRNA ribonucleoprotein complexes compared to IgG immunoprecipitates (Fig. 4f). These data suggest that miR-383 can directly target PTTG3P in HCC.

### PTTG3P/miR-383/CCND1 or PARP2 axis modulates HCC cell growth, migration and invasion

**Fig. 5:**
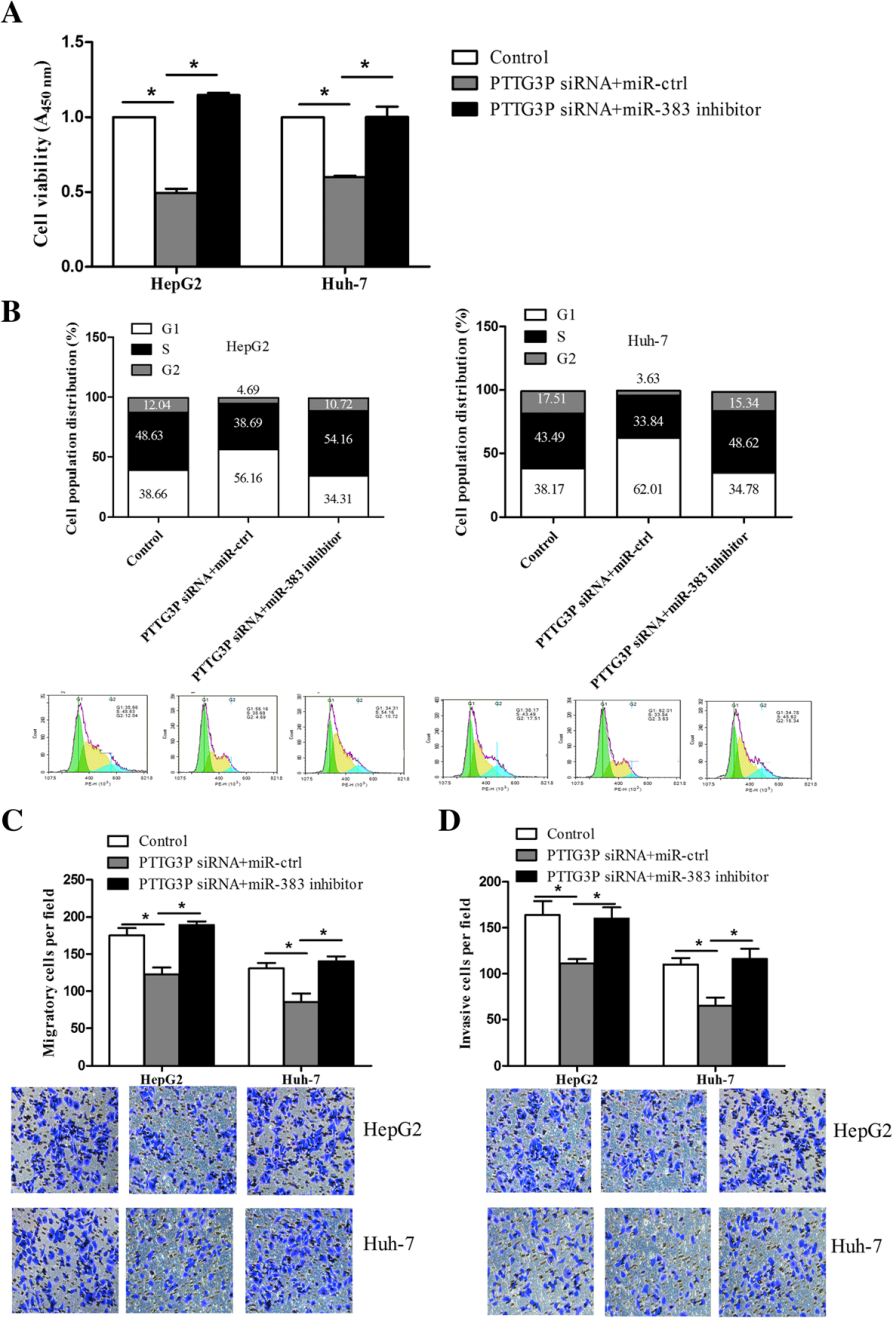
PTTG3P/miR-383 axis in the phenotypes of HCC cells. HepG2 and Huh-7 cells were co-transfected with PTTG3 siRNA and miR-383 inhibitor, or corresponding controls, and then subjected to CCK-8 assay to detect the cell viability (**a**), or to flow cytometry to detect the cell cycle distribution (**b**), or to Transwell migration (**c**) and invasion (**d**) assays to detect the changes in the migratory and invasive abilities of HCC cells. The color code in 5B represented the G1 stage, S stage and G2 stage, respectively (Left to right). The results were shown as mean ± SD from three independent experiments and one of the representative results was shown. Each experiment was performed in triplicate. *P < 0.05

Considering that miR-383 was a target of PTTG3P in HCC cells, we tried to elucidate whether the potential ceRNA mechanism among LncRNA PTTG3p, miR-383 and miR-383’s targets do exist. Firstly, we performed rescue experiments to observe the roles of PTTG3P/miR-383 in regulating HCC cells growth, migration and invasion. HepG2 and Huh-7 cells were co-transfected with PTTG3P siRNA and miR-383 inhibitor or controls, and subjected to functional experiments. As results shown in Fig. 5a, we found that inhibition of miR-383 ameliorated the anti-growth of PTTG3P knockdown, compared to control group. The cell cycle analysis indicated that inhibition of miR-383 promoted G1/S transition that was suppressed by PTTG3P knockdown (Fig. 5b). Similarly, the data from Transwell migration and invasion assays showed that miR-383 inhibition enhanced the number of migratory and invasive cells which was reduced by PTTG3P knockdown (Fig. 5c and d). These data illustrate that miR-383 inhibition abolishes the anti-growth and anti-metastasis activities of PTTG3P knockdown in HCC cells.

**Fig. 6:**
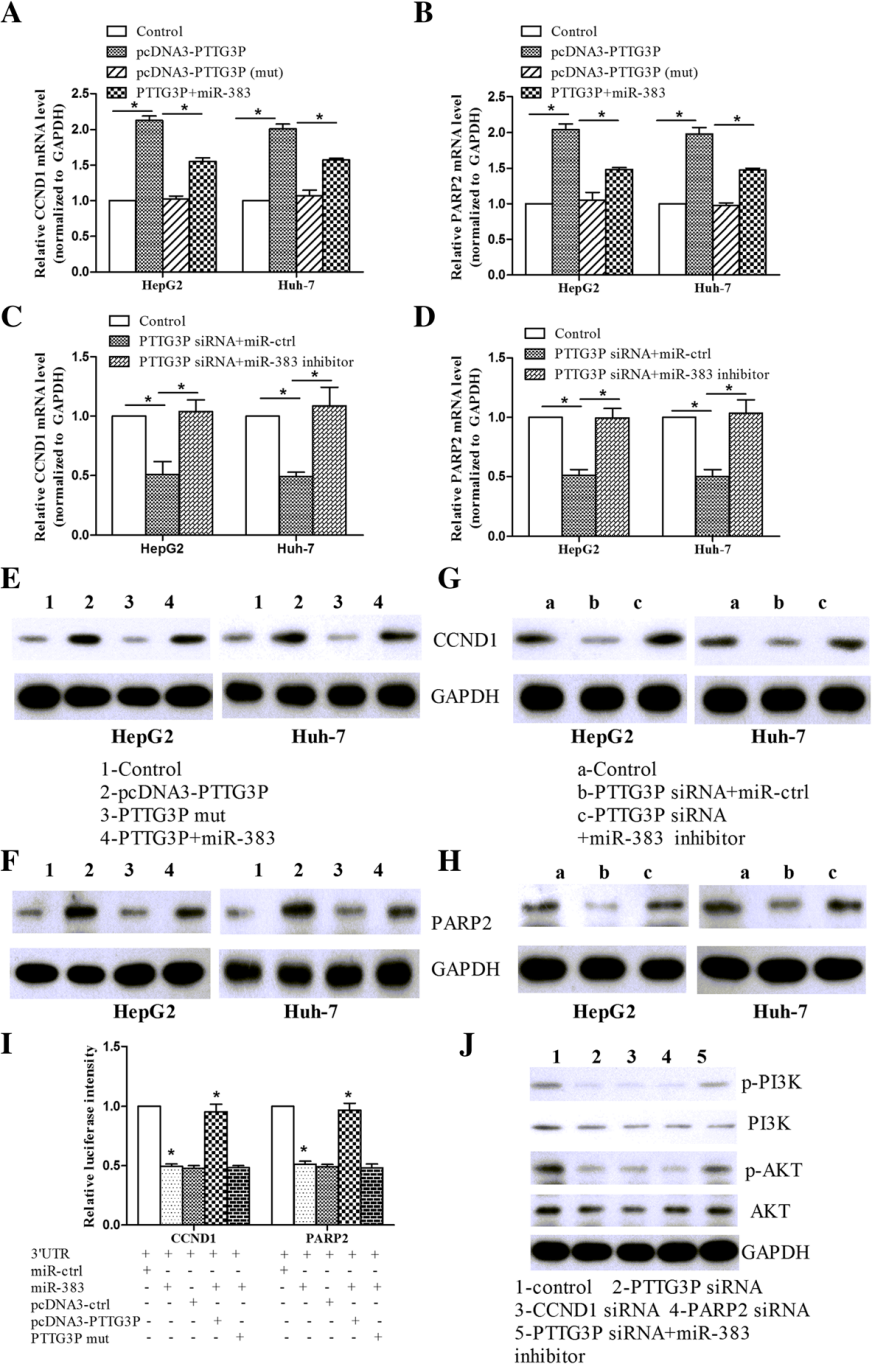
PTTG3P affects the expression of miR-383 targets in HCC cells. **a**, **b** HepG2 and Huh-7 cells were transfected with PTTG3P, or mutant PTTG3P, or PTTG3P and miR-383, or control vector, and then qPCR was used to detect CCND1 (**a**) and PARP2 (**b**) expression. **c**, **d** HepG2 and Huh-7 cells were transfected with PTTG3P siRNA, or PTTG3P siRNA and miR-383 inhibitor, or controls, and then qPCR was performed to detect CCND1 (**c**) and PARP2 (**d**) expression. **e**, **f** HepG2 and Huh-7 cells were transfected with PTTG3P, or mutant PTTG3P, or PTTG3P and miR-383, or control vector, and then Western blot assay was used to detect CCND1 (**e**) and PARP2 (**f**) expression. **c**, **d** HepG2 and Huh-7 cells were transfected with PTTG3P siRNA, or PTTG3P siRNA and miR-383 inhibitor, or controls, and then qPCR was performed to detect CCND1 (**g**) and PARP2 (**h**) expression. **i** Luciferase reporter assay was performed to detect CCND1 3’UTR or PARP2 3’UTR intensity in the cells co-transfected with target 3’UTR and miR-383 and PTTG3P, or mutant PTTG3P. **j** Western blot assay was performed to analyze the phosphorylation of PI3K/Akt signaling pathway in cells transfected with siRNA against PTTG3P, or CCND1, or PARP2, or PTTG3P siRNA and miR-383 inhibitor. The results were shown as mean ± SD from three independent experiments and one of the representative results was shown. Each experiment was performed in triplicate. *P < 0.05

We next determined whether PTTG3P can modulate the expression of miR-383 targets in HCC cells. CCND1 and PARP2 are validated miR-383 targets as previously described [24, 25]. As shown in Fig. 6a, we found that PTTG3P overexpression increased CCND1 mRNA levels in HepG2 and Huh-7 cells, while miR-383 overexpression can restore CCND1 expression increased by PTTG3P. However, mutant PTTG3P did not affect CCND1 expression when the binding sites of miR-383 were mutated. Similar tendency was shown in PARP2 expression levels (Fig. 6b). By contrast, knockdown of PTTG3P reduced CCND1 and PARP2 expression levels, while miR-383 inhibition can abrogate the inhibitory effect of PTTG3P on CCND1 and PARP2 (Fig. 6c and d). The protein levels of CCND1 and PARP2 were in line with that of CCND1 and PARP2 mRNA (Fig. 6e-h, Additional file 1: Figure S1A-D). The luciferase assay indicated that miR-383 reduced the luciferase intensity controlled by CCND1 3’UTR, while PTTG3P but not mutant PTTG3P abolished the inhibitory roles of miR-383 in CCND1 3’UTR intensity (Fig. 6i). In line with the effect of miR-383 on CCND1, similar results were shown in PARP2-related assays. Finally, we found that siRNA against PTTG3P or CCND1 or PARP2 inhibited the phosphorylation of PI3K and Akt, while miR-383 inhibition was co-transfected, the inhibitory roles of PTTG3P knockdown were abolished (Fig. 6j, Additional file 1: Figure S1E).

## Discussion

Accumulating evidence indicates that aberrant LncRNA expression is related to HCC tumorigenesis, such as HOTAIR [26], MALAT1 [27] and TUG1 [28]. In this study, we found that pseudogene-derived LncRNA PTTG3P was upregulated in HCC tissues and cells, and high PTTG3P level was related to tumor size and metastasis. Knockdown of PTTG3P suppressed cell cycle progression, cell proliferation, migration and invasion, and promoted cell apoptosis, functioning as an oncogene. In line with some data obtained in our study, previous research shows that PTTG3P is highly expressed in gastric cancer tissues, and promotes cell proliferation, migration and invasion in vitro and in vivo, functioning as an independent negative prognostic biomarker [20]. Another study indicates that PTTG3P is upregulated in HCC tissues and promotes cell growth and metastasis through activating PI3K/Akt pathway [21]. However, the miRNA involved in PTTG3P-mediated HCC development is not revealed.

As gene regulators, LncRNAs modulate gene expression via a variety of mechanism, and one of the main mechanism is to harbor miRNA to serve as a sponge to up or downregulate miRNA expression. In this study, the bioinformatics analysis indicated the potential interaction between miR-383 and PTTG3P. Luciferase assay validated that PTTG3P was a direct target of miR-383, together with the RNA pull-down and RIP assays, and miR-383 was downregulated in HCC tissues. MiR-383 modulated the functions of PTTG3P in tumor malignant phenotypes. LncRNA plays crucial roles partially via acting as a ceRNA and modulating the expression of miRNA targets as previously described [29–31]. Accordingly, as a sponge of miR-383, PTTG3P upregulated the mRNA and protein expressions of miR-383 targets, CCND1 and PARP2, while mutant PTTG3P (the binding sites between miR-383 and PTTG3P were mutated) did not affect CCND1 and PARP2 expression levels, as well as the luciferase intensity. CCND1 and PARP2 are reported to be involved in tumor development and act as oncogenes. For example, CCND1 promotes the colon cancer development via PI3K/Akt pathway [32]. MiR-503 suppresses cell proliferation, migration and invasion via suppressing CCND1 expression in breast cancer [33] and ESCC [34]. MiR-383 suppresses cervical cancer cell proliferation, invasion and metastasis by inhibiting PI3K/Akt pathway via the downregulating of PARP2 [25]. In line with the findings in the above stidues, our data showed that knockdown of CCND1 and PARP2 suppressed the phophorylation of PI3K and Akt, similar to that of PTTG3P knockdown, while miR-383 inhibition can restore the inhibitory effects of PTTG3P knockdown on OI3K/Akt pathway.

## Conclusion

In summary, we identified a PTTG3P-miR-383-CCND1/PARP2 axis in HCC pathogenesis. PTTG3P acted as an oncogenic lncRNA to promote HCC development through upregulating CCND1 and PARP2 as well as PI3K/Akt pathway via sponging miR-383. The data suggest that PTTG3P may serve as a potential therapeutic target in HCC.

## Additional files

Additional file 1: **Figure S1.** Relative protein expression of CCND1 (A, C) and PARP2 (B, D) as well as phosphorylation level of PI3K and Akt (E). *P<0.05. (TIF 763 kb)

Additional file 2: **Figure S2.** The percentage of apoptotic cells in HepG2 and Huh-7 cells after treatment with PTTG3P siRNA or control. *P<0.05. (TIF 104 kb)

## Data Availability

All data generated or analyzed during this study are included in this published article or are available from the corresponding author on reasonable request.

## Abbreviations

3’UTR: 3’untranslated region
CCK-8: Cell Counting Kit-8
CCND1: Cyclin D1
FBS: Fetal bovine serum
HCC: Hepatocellular carcinoma
HRP: Horseradishperoxidase
LncRNA: Long non-coding RNA
miRNA: microRNA
PARP2: Poly ADP-ribose polymerase 2
PTTG3P: Pituitary tumor-transforming 3
