# Supplementary figures and images for: Long non-coding RNA PTTG3P functions as an oncogene by sponging miR-383 and up-regulating CCND1 and PARP2 in hepatocellular carcinoma
Source: BMC Cancer. 2019 Jul 24;19:731. doi: 10.1186/s12885-019-5936-2 (PMC6657059; doi:10.1186/s12885-019-5936-2)

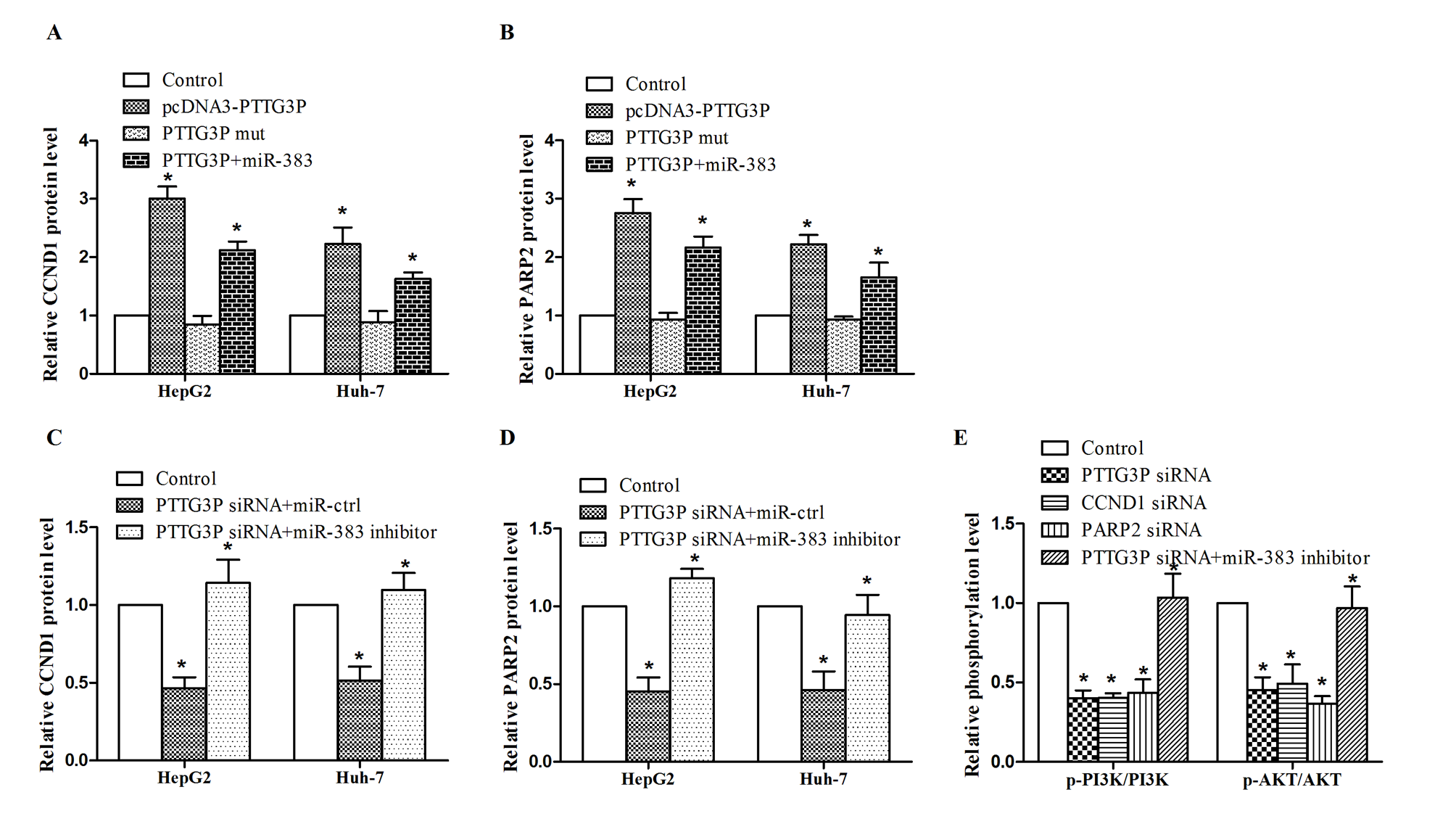

Supplement: Supplementary file 1 — Figure S1. Relative protein expression of CCND1 (A, C) and PARP2 (B, D) as well as phosphorylation level of PI3K and Akt (E). *P<0.05. (TIF 763 kb) [file 12885_2019_5936_MOESM1_ESM.tif]

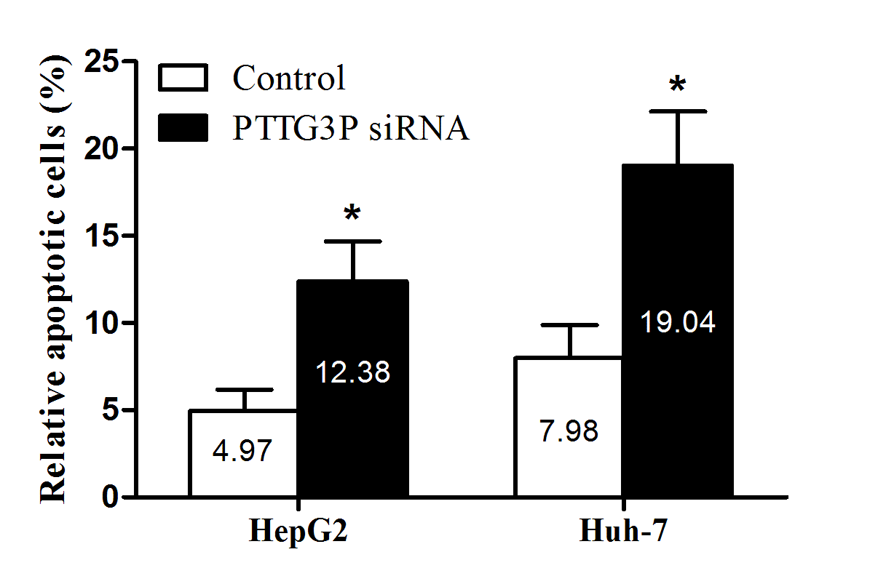

Supplement: Supplementary file 2 — Figure S2. The percentage of apoptotic cells in HepG2 and Huh-7 cells after treatment with PTTG3P siRNA or control. *P<0.05. (TIF 104 kb) [file 12885_2019_5936_MOESM2_ESM.tif]
